# Pathogenic germline variants in *SMARCA4* and further cancer predisposition genes in early onset ovarian cancer

**DOI:** 10.1002/cam4.6214

**Published:** 2023-06-22

**Authors:** Natalie Herold, Johanna Schmolling, Corinna Ernst, Beyhan Ataseven, Britta Blümcke, Birgid Schömig‐Markiefka, Sebastian Heikaus, Uwe‐Jochen Göhring, Christoph Engel, Björn Lampe, Kerstin Rhiem, Philipp Harter, Jan Hauke, Rita K. Schmutzler, Eric Hahnen

**Affiliations:** ^1^ Center for Familial Breast and Ovarian Cancer, Center for Integrated Oncology (CIO), Medical Faculty University of Cologne and University Hospital Cologne Cologne Germany; ^2^ Department of Gynecology & Gynecologic Oncology Kliniken Essen‐Mitte (KEM) Essen Germany; ^3^ Medical School and University Medical Center East Westphalia‐Lippe, Klinikum Lippe, Academic Department of Gynecology, Gynecologic Oncology and Obstetrics Bielefeld University Detmold Germany; ^4^ Institute of Pathology University Hospital Cologne Cologne Germany; ^5^ Department of Pathology Kliniken Essen‐Mitte (KEM) Evang. Huyssens‐Stiftung/Knappschaft GmbH Essen Germany; ^6^ Department of Gynecology and Obstetrics Johanniter Krankenhaus Bonn Germany; ^7^ Institute for Medical Informatics, Statistics and Epidemiology University of Leipzig Leipzig Germany; ^8^ Department of Gynecology and Obstetrics Diakonie Kaiserswerth Düsseldorf Germany

**Keywords:** BRCA mutations, cancer risk factors, gynecological oncology, hereditary cancer, ovarian cancer, SCCOHT, *SMARCA4*

## Abstract

To assess the role of germline pathogenic variants (PVs) in *SMARCA4* and further established ovarian cancer (OC) predisposition genes in early onset OC, we investigated a clinical cohort of 206 unrelated OC index patients with an age at diagnosis of OC ≤40 years using an extended panel of 24 (candidate) cancer predisposition genes. PVs in established OC predisposition genes were most frequent in patients with high grade serous OC (21/62, 33.9%), comparatively rare in patients with epithelial OC other than high grade serous (5/74, 6.8%) or borderline ovarian tumours (2/39, 5.1%) and absent in mucinous OC (0/27). We demonstrate that germline PVs in *SMARCA4* unlikely predispose for early onset OC other than SCCOHT.

*SMARCA4*, a gene associated with the small cell carcinoma of the ovary, hypercalcemic type (SCCOHT), appears to be the sole ovarian cancer (OC) predisposition gene associated with a very early age at diagnosis (≤40 years). To assess the role of germline pathogenic variants (PVs) in *SMARCA4* and further established OC predisposition genes in early onset OC, we investigated a clinical cohort of 206 unrelated index patients using an extended panel of 25 (candidate) cancer predisposition genes. PVs in established OC predisposition genes were most frequent in patients with high grade serous OC (21/62, 33.9%), rare in patients with other epithelial OC (5/74, 6.8%) or borderline ovarian tumors (2/39, 5.1%). Their absence in mucinous OC (0/27) supports the notion that this entity represents a tumor phenotype not associated with PVs in established OC predisposition genes. PVs in *SMARCA4* were restricted to SCCOHT and unlikely predispose for early onset OC other than SCCOHT.

According to the recommendations of the German Consortium for Hereditary Breast and Ovarian Cancer (GC‐HBOC), all women diagnosed with epithelial ovarian cancer (OC) before the age of 80 years should have germline genetic testing of *BRCA1/2* and other OC predisposition genes, irrespective of their tumor histology or family cancer history.^1^ The American Society of Clinical Oncology (ASCO) recommends genetic testing for all women diagnosed with epithelial OC, irrespective of their age at diagnosis (AAD), tumor histology, or family cancer history. It was suggested by the ASCO that multigene panel testing should cover, at minimum, ten selected OC predisposition genes (*BRCA1*, *BRCA2*, *BRIP1*, *MLH1*, *MSH2*, *MSH6*, *PMS2*, *PALB2*, *RAD51C*, and *RAD51D*).^2^ The probability to detect germline pathogenic variants (PVs) in the ten selected OC predisposition genes is largely dependent on the family cancer history, tumor histology, and AAD. Two large clinical cohort investigations revealed an overall mean AAD of OC of approximately 60 years, while patients with PVs in *BRCA1* and the mismatch repair genes *MSH2* and *MSH6* demonstrate a statistically significant earlier mean AAD than OC patients without.^3^, ^4^ Germline PVs in the selected OC predisposition genes other than *BRCA1*, however, were rare in OC patients with an early AAD of OC ≤40 years.^4^ In addition to the ten selected OC predisposition genes, germline PVs causing a loss of *SMARCA4* gene function predispose for the rare small cell carcinoma of the ovary, hypercalcemic type (SCCOHT), primarily affecting females under 40 years with an average age of onset of 24–30 years.^5^, ^6^, ^7^ Based on the published literature, *SMARCA4* appears to be the sole OC predisposition gene associated with an AAD ≤40 years. Germline PVs in OC predisposition genes rarely associate with one tumor phenotype only. For example, germline PVs in *BRCA1* associate primarily with high grade serous OC tumor phenotype, but also with high grade endometrioid and clear cell OC, though with a lower PV prevalence.^4^ Thus, our study has two objectives. First, we aimed to assess the potential role of germline PVs in *SMARCA4* along with an extended panel of 24 additional (candidate) cancer predisposition genes in a clinical cohort of 206 OC patients (including three SCCOHT index patients) with an AAD ≤40 years. Second, we aimed to characterize families with *SMARCA4* PVs to gain more insights into *SMARCA4‐*dependent cancer predisposition and progression.

Key characteristics of the overall study sample according to the tumor classification of the WHO (https://tumourclassification.iarc.who.int) are given in Table 1. Targeted next‐generation sequencing (NGS) analyses and variant classification were performed in a routine diagnostic setting at the Center for Familial Breast and Ovarian Cancer, Cologne, Germany (Methods in the supplement). Of the 25 genes investigated, 12 were defined as established OC predisposition genes (*ATM*, *BRCA1*, *BRCA2*, *BRIP1*, *MLH1*, *MSH2*, *MSH6*, *PALB2*, *PMS2*, *RAD51C*, *RAD51D*, and *SMARCA4*).^3^, ^10^, ^11^, ^12^ In the overall study sample, 22 carriers of PVs in the *BRCA1/2* genes were identified (22/206, 10.7%), of which 21 carried PVs in *BRCA1*. All but two *BRCA1/2* mutation carriers developed high grade serous tumors and a positive *BRCA1* mutation status was statistically significantly associated with this subtype (*p* < 10^−8^, Fisher's exact test, Methods in the supplement). The remaining two patients, both *BRCA1* PV carriers, developed an endometrioid and a clear cell tumor (Table S1). A positive *BRCA1* PV status was statistically significantly associated with a later AAD (mean 37.5 years, range 31–40 years) compared with OC patients without *BRCA1* PVs (mean 32.7 years, range 13–40 years, *p* = 0.003, *t*‐test). We detected no *BRCA1/2* PV in patients with an AAD ≤30 years (0/83), 3 *BRCA1* PVs in patients with an AAD between >30 and ≤35 years (3/61, 4.9%), and 18 *BRCA1* and 1 *BRCA2* PVs in patients with an AAD between >36 and ≤40 years (19/62, 30.6%). This is consistent with previous findings that germline PVs in *BRCA1* prevail in OC patients with an AAD ≤40 years and PVs in both *BRCA1* and *BRCA2* are virtually absent in OC patients with an AAD ≤30 years.^1^, ^4^ With a cumulative OC risk of 0.3% until the age of 40 years for *BRCA2* PV carriers (vs 1.8% for *BRCA1* PV carriers), our study sample is likely depleted for *BRCA2* PV carriers.^13^ A total of ten OC patients, all *BRCA1/2* negative, carried PVs in established non‐*BRCA1/2* OC predisposition genes (10/206, 4.9%). PVs in the *ATM*, *RAD51C*, and *SMARCA4* genes were identified in two patients each, and in *BRIP1*, *MLH1*, *MSH2*, and *PMS2* in one patient each (Table 1, Table S1). PVs in *BRCA1/2* and established non‐*BRCA1/2* OC predisposition genes were found across all tumor phenotypes, except mucinous carcinoma. PVs in the *SMARCA4* gene were restricted to patients with SCCOHT. Two of the three unrelated SCCOHT index patients carried germline PVs in the *SMARCA4* gene (patients #1 and #2), and complementary genetic tumor analyses suggested a loss of the *SMARCA4* wildtype alleles. The same holds true for the affected sister of patient #2, in which the SCCOHT tumor phenotype was confirmed in retrospect. The third patient with a SCCOHT tumor phenotype (patient #3) showed a biallelic somatic *SMARCA4* inactivation (see supplement for detailed medical cancer history of all three SCCOHT families, including genetic test results). Witkowski et al. demonstrated that women with SCCOHT and germline PVs present at a significantly younger age than those without.^14^ In line with this observation, we described here three *SMARCA4* germline PV carriers out of two families (patient #1, patient #2 and her sister) showing an AAD of 25, 26, and 27 years, respectively, while a SCCOHT patient with an exclusively somatic *SMARCA4* inactivation (patient #3) had an AAD of 35 years. It has been reported that regardless of tumor stage, most SCCOHT patients relapse and die of disease within a comparatively short period of time, with an estimated one‐year overall survival rate of 50% and a five‐year overall survival rate of less than 10%.^15^ A review of 47 cases revealed a median overall survival of 14.9 months only, which increased to 35.3 months when SCCOHT was diagnosed at tumor stage I.^5^ Young et al. reported that 14 of 42 patients with stage IA disease remained well and free of disease 1–13 (average 5.7) years postsurgery.^7^ In our investigation, two patients with a stage IA disease and a germline PV in *SMARCA4* remained cancer‐free for 7 years (patient #1) and 5 years (patient #2), respectively, following platinum‐based multi‐agent regimen. Future clinical trials may assess whether SCCOHT patients with germline PVs in *SMARCA4* have a better therapy outcome compared with SCCOHT patients without germline PVs in *SMARCA4*.

**TABLE 1:** Key characteristics of the study sample.

Histological OC subtype	*n* (%)	Mean AAD (range) in years	Positive cancer family history (%)	PV in gene (*n*)^b^
Overall	206 (100.0)	31.3 (13–40)	100 (48.5)	/
High grade serous^a^	62 (30.1)	33.1 (21–40)	27 (43.5)	*BRCA1 (19)*, *BRCA2 (1)*, *BRIP1 (1)*, *CHEK2 (2)*, *RAD50 (1)*
Borderline ovarian tumor	39 (18.9)	29.4 (13–40)	26 (66.7)	*ATM (2)*
Mucinous^a^	27 (13.1)	28.0 (19–39)	15 (55.6)	*MUTYH (1)*
Low grade serous	26 (12.6)	31.0 (18–40)	8 (30.8)	*FANCM (1)*, *RAD51C (1)*
Endometroid^a^	15 (7.3)	34.7 (26–40)	3 (20.0)	*BRCA1 (1)*, *MLH1 (1)*
Missing	15 (7.3)	35.5 (25–40)	11 (73.3)	*FANCM (1)*, *MUTYH (1)*, *MSH2 (1)*
Others/unspecified	13 (6.3)	29.2 (16–36)	6 (46.2)	*RAD51C (1)*
Clear cell	6 (2.9)	30.0 (19–39)	3 (50.0)	*BRCA1 (1)*, *FANCM (1)*, *PMS2 (1)*, *TP53* ^c^ *(1)*
SCCOHT	3 (1.5)	29.3 (26–35)	1 (33.3)	*SMARCA4 (2)*

Note: Unrelated index patients with an age at diagnosis (AAD) of ovarian cancer (OC) ≤ 40 years and any epithelian histological OC subtype, borderline ovarian tumors and SCCOHT were eligible for this retrospective investigation. Patients with germ cell tumors of the ovary were excluded. A total of 206 index patients recruited between 2008 and 2020 by the Cologne Center for Familial Breast and Ovarian Cancer and cooperating Gynaecological Cancer Centers, met these criteria; 196 patients had a personal history of OC only; 10 patients had a personal history of OC and breast cancer (BC). We retrieved histopathological data of the OC, personal and familial OC/BC history from the centralized patient database of the German Consortium Hereditary Breast‐ and Ovarian Cancer (GC‐HBOC).^8^ A positive cancer family history was defined as the existence of at least one relative with BC or OC. Written informed consent was obtained from all patients, and ethical approval was granted by the ethics committee of the University of Cologne (07–048). Ninety‐six OC patients were included in a previous study focusing on in silico tools for the prediction of germline copy number variations.^9^

^a^ The mucinous tumor phenotype was statistically significantly associated with an earlier AAD (mean 28.0 years, range 19–39 years) than OC patients with other tumor phenotypes (mean 31.5 years, range 13–40 years, adjusted *p* = 0.02, two‐sided *t*‐test). Two tumor phenotypes were statistically significantly associated with later AAD, namely the high grade serous (mean AAD 33.1 vs. 30.0 years, range 21–40 vs. 13–40 years; adjusted *p* = 0.009) and the endometrioid tumor phenotype (mean AAD 34.7 vs. 30.7 years, range 26–40 vs. 13–40 years; adjusted *p* = 0.03).

^b^ We identified no germline pathogenic variants (PVs) in *BARD1*, *CDH1*, *FAM175A*, *MRE11A*, *MSH6*, *NBN*, *PALB2*, *PTEN*, *RAD51D*, *STK11*, and *XRCC2*. Nine patients, all negative for PVs in established OC predisposition genes, carried PVs in candidate cancer predisposition genes not clearly associated with OC (*CHEK2*, *FANCM*, *MUTYH*, *RAD50*, *TP53*, Table S1).

^c^ The *TP53* PV c.920‐1G > A was detected in blood‐derived DNA with a variant fraction (VF) of 47% (read coverage: 952) and with a VF of 97% (read coverage: 732) in tumor‐derived DNA. Thus, this variant identified in a patient with an AAD of 19 years most likely represents a germline PV.

The by far highest germline PV prevalence in established OC predisposition genes was observed in patients with high grade serous OC (21/62, 33.9%). In contrast, germline PVs in established OC predisposition genes were rare or even absent in patients with borderline (2/39, 5.1%), low grade serous (1/26, 3.8%), and mucinous (0/27) ovarian tumors. The mucinous tumor phenotype was highly enriched in our study sample compared with study samples unselected for AAD, in which the proportion of this tumor phenotype was reported to be approximately 3%.^16^ Norquist et al. described 16 out of 1915 OC patients with a mucinous tumor phenotype (0.8%).^4^ Lilyquist et al. described 94 out of 1637 patients with OC showing a mucinous tumor phenotype (5.7%).^3^ Of note, the study sample described by Norquist et al. included only a small proportion of OC patients with an AAD ≤40 (51/1915; 2.7%) compared with Lilyquist et al. (748/7769; 9.6%). In our study sample of OC patients with an AAD ≤40, the mucinous tumor phenotype accounted for 13.1% (27/206). Thus, the proportion of the mucinous tumor phenotype appeared to be inversely correlated with the mean AAD of the overall study sample, which is consistent with the observation that 26% of all patients with mucinous OC are younger than 44 years at presentation.^16^ An intriguing finding is that in our study sample none of the 27 patients with mucinous OC carried a germline PV in the established OC predisposition genes. The same holds true for the 16 patients with mucinous OC described by Norquist et al., and the nine patients with mucinous OC described in our previously reported observational AGO‐TR1 trial.^1^ Thus, our findings support the notion that mucinous OC represents a separate disease entity not associated with PVs in *BRCA1/2* ^16^ or other established OC predisposition genes.^4^

## AUTHOR CONTRIBUTIONS

**Natalie Herold:** Data curation (equal); investigation (equal); writing – original draft (equal); writing – review and editing (equal). **Johanna Schmolling:** Data curation (equal); resources (equal); writing – review and editing (equal). **Corinna Ernst:** Data curation (lead); writing – review and editing (equal). **Beyhan Ataseven:** Resources (equal); writing – review and editing (equal). **Britta Bluemcke:** Data curation (equal); writing – review and editing (equal). **Birgid Schömig‐Markiefka:** Resources (equal); writing – review and editing (equal). **Sebastian Heikaus:** Resources (equal); writing – review and editing (equal). **Uwe‐Jochen Goehring:** Resources (equal); writing – review and editing (equal). **Christoph Engel:** Resources (equal); software (equal); writing – review and editing (equal). **Bjoern Lampe:** Resources (equal); writing – review and editing (equal). **Kerstin Rhiem:** Resources (equal); writing – review and editing (equal). **Philipp Harter:** Resources (equal); writing – review and editing (equal). **Jan Hauke:** Resources (equal); writing – review and editing (equal). **Rita K. Schmutzler:** Funding acquisition (equal); resources (equal); supervision (equal); writing – review and editing (equal). **Eric Hahnen:** Conceptualization (lead); data curation (equal); formal analysis (equal); funding acquisition (equal); investigation (equal); methodology (equal); project administration (equal); supervision (equal); writing – review and editing (equal).

## FUNDING INFORMATION

The GC‐HBOC is supported by the German Cancer Aid (grant no 110837 and grant no 70114178, coordinator: Rita K. Schmutzler, Cologne) and the Federal Ministry of Education and Research (BMBF), Germany (grant no 01GY1901). Genetic analyses were supported by the Köln Fortune Program, Faculty of Medicine, University of Cologne, Germany. The funding sources had no role in the design and conduct of the study; collection, management, analysis, and interpretation of the data; preparation, review, or approval of the manuscript; nor in the decision to submit the manuscript for publication. We acknowledge support for the Article Processing Charge from the German Research Foundation (DFG, 491454339).

## Supporting information


**Table S1:** Patient characteristics and germline test results.

**Appendix S1:** Supporting Information.

## Data Availability

Data available in article supplementary material.
