# Supplementary material for: Pathogenic germline variants in SMARCA4 and further cancer predisposition genes in early onset ovarian cancer
Source: Cancer Med. 2023 Jun 22;12(14):15256–60. doi: 10.1002/cam4.6214 (PMC10417158; doi:10.1002/cam4.6214)
Supplement: Supplementary file 2 — Appendix S1: Supporting Information. [file CAM4-12-15256-s002.docx]

**Pathogenic germline variants in *SMARCA4* and further cancer predisposition genes in early onset ovarian cancer**

**SUPPLEMENTARY METHODS**

**Next-generation sequencing (NGS)**

Targeted NGS was performed using a customized hybrid capture gene panel (TruRisk® gene panel, Agilent SureSelect, QXT protocol) on an Illumina NextSeq 500 sequencing device (Illumina, San Diego, USA). The hybridization capture-based NGS method was suitable for the analysis of DNA derived from either blood or formalin-fixed paraffin-embedded (FFPE) tumour samples. Isolation of DNA derived from blood and FFPE tumour samples was performed using standard techniques. The TruRisk® gene panel covered the entire coding regions and exon-flanking sequences (±15 nt) of *SMARCA4* (NM_001128844.1) and 24 further (candidate) cancer predisposition genes (*ATM,* NM_000051.3; *BARD1,* NM_000465.3; *BRCA1*, NM_007294.3; *BRCA2*, NM_000059.3; *BRIP1,* NM_032043.2; *CDH1,* NM_004360.4; *CHEK2,* NM_007194.3; *FAM175A*, NM_139076.2; *FANCM*, NM_000136.2; *MLH1,* NM_000249.3; *MRE11A,* NM_005591.3; *MSH2,* NM_000251.2; *MSH6,* NM_000179.2; *MUTYH*, NM_001128425.1; *NBN,* NM_002485.4*; PALB2,* NM_024675.3; *PMS2,* NM_000535.6; *PTEN,* NM_000314.6*; RAD50,* NM_005732.3; *RAD51C,* NM_058216.2; *RAD51D,* NM_002878.3; *STK11,* NM_000455.4; *TP53,* NM_000546.5, *XRCC2*, NM_005431.1)*.* Of the 25 genes investigated, 12 were defined as established OC predisposition genes (*ATM*, *BRCA1*, *BRCA2*, *BRIP1*, *MLH1, MSH2*, *MSH6*, *PALB2*, *PMS1*, *RAD51C*, *RAD51D* and *SMARCA4*).

**Next-generation sequencing (NGS) data analysis and variant classification**

The Alamut Visual version 2.13 analysis software tool (Interactive Biosoftware, Rouen, France) was applied for variant annotation and integration of current ClinVar classifications. Variant classification was performed using the criteria of the German Consortium for Hereditary Breast and Ovarian Cancer (GC-HBOC) for the classification of germline sequence variants in predisposition genes for hereditary BC and OC (Wappenschmidt et al., 2020), which are based on the guidelines by the Evidence-Based Network for the Interpretation of Germline Mutant Alleles (ENIGMA, www.enigmaconsortium.org, accessed December 2019) and the American College of Medical Genetics and Genomics (ACMG) (Richards et al., 2015). As proposed by the International Agency for Research on Cancer (IARC), a five-tier classification system was applied (Plon et al., 2008). This classification system defines germline variants as pathogenic (class 5), likely pathogenic (class 4), variant of uncertain significance (VUS, class 3), likely benign (class 2), or benign (class 1). Protein-truncating variants (PTVs) were defined as nonsense, frameshift, or essential splice site variants affecting the invariant splice sites or the last nucleotide of an exon. Pathogenic variants (PVs) include (likely) pathogenic PTVs and (likely) pathogenic missense variants. All PVs were verified by Sanger sequencing. For the prediction of copy number variations (CNVs) in blood-derived DNA, we employed the CE-IVD-marked Sophia Genetics DDM pipeline v3.4.0–4.6.2 (Sophia Genetics, Saint-Sulpice, Switzerland), as described previously (Lepkes et al., 2021). Predicted CNVs were verified by multiplex ligation-dependent probe amplification (MLPA) using SALSA^®^ MLPA^®^ kits (MRC Holland, Amsterdam, The Netherlands).

**Statistical analyses**

Statistical analyses were performed using R v3.6.3. All tests were two-sided with P values < 0.05 considered statistically significant. Adjustments for multiple testing correction were performed using the Benjamini-Hochberg approach.

**MEDICAL CANCER HISTORY SCCOHT FAMILIES**

At the age of 26 years, **patient #1** presented with a SCCOHT of 15 cm and a hypercalcaemia of 3.84 mmol/l (norm 2.15-2.5 mm/l). Tumour stage after fertility preserving surgery was pT1a, pN0 (0/49), G3, FIGO IA according to the International Federation of Gynaecology and Obstetrics (Prat and Oncology, 2015). Adjuvant chemotherapy consisted of 4 cycles of etoposide and cisplatin. Until 08/2022, at 32 years, there was no evidence for recurrent disease. Analysis of blood-derived DNA of patient #1 revealed a heterozygous germline PV in exon 11 of the *SMARCA4* gene (c.1648dupC, p.(Leu550Profs*14)). No germline PV in further established OC predisposition genes was observed. Genetic analysis of the tumour-derived DNA revealed a variant fraction (VF) of the c.1648dupC variant of 83% (read coverage: 662), suggesting loss of the wildtype allele. No additional somatic variants in the *SMARCA4* gene were observed. The three-generation pedigree showed no further patients with BC or OC. Predictive testing of the unaffected mother of patient #1 by Sanger sequencing identified her as a heterozygous carrier of the germline PV c.1648dupC. Risk reducing salpingo-oophorectomy at the age of 57 showed no evidence for malignancy.

At the age of 27 years, **patient #2** presented with a solid pelvic mass of 3.7 cm and a hypercalcaemia of 2.62 mmol/l. Histopathology revealed typical signs of SCCOHT, confirmed by loss of SMARCA4 nuclear immunoreactivity in reference pathology consultation. The tumour stage was pT1a, pN0 (0/40), G3, FIGO IA. Adjuvant chemotherapy with cisplatin, doxorubicin, and etopositde cyclophospaamide (PAVEP regimen) was applied, followed by high dose chemotherapy with carboplatin, vepeside, cyclophosphamide (CARBOPEC) and stem-cell support after complete remission (Pautier et al., 2007). Until 08/2022 (age 33 years) there was no evidence for recurrent disease. Analysis of blood-derived DNA revealed a heterozygous PV in exon 5 of the *SMARCA4* gene (c.810dupC, p.(Gly271Argfs*16)). No germline PVs in further established OC predisposition genes were observed. Genetic analysis of the tumour-derived DNA revealed a VF of the germline c.810dupC variant of 45% (read coverage: 166). In addition, a somatic PV in exon 16 was observed (c.2438+1G>A, splice donor), with a VF of 37% (read coverage: 586). The three-generation pedigree showed one further patient with OC and no patient with BC. The older sister of patient #2 was previously diagnosed with OC at the age of 25 years in 2011. She presented with abdominal pain and an abdominal mass one week after vaginal birth. MRI showed a pelvic mass of 20x12x17 cm with ascites. Initial histopathological analysis revealed an undifferentiated OC (G4) of the right ovary with microscopical peritoneal metastases. She died weeks after diagnosis (pT3a pN1 (2/58) pL1 pV0, FIGO IIIC). Retrospective analysis of tumour-derived DNA confirmed the germline PV identified in her younger sister (c.810dupC, p.(Gly271Argfs*16)), with a VF of 38% (read coverage: 123). In addition, a somatic PV in exon 6 was observed (c.1111dupG, p.(Glu371Glyfs*16)), with a VF of 35% (read coverage: 238). In knowledge of the *SMARCA4* germline PV, a reference pathological examination was performed in 2021, showing a loss of SMARCA4 nuclear immunoreactivity. The tumour cells were positive for P16, WT, and negative for CD10 and Calretinin, a histopathological pattern that matches the large cell variant of SCCOHT (Lu and Shi, 2019, Young et al., 1994, Ferlicot et al., 1998). At the age of 62 years, predictive genetic testing of the unaffected mother of patient #2 by NGS identified her as a heterozygous carrier of the germline *SMARCA4* PV c.1648dupC. She underwent unilateral adnexectomy at the age of 30 and contralateral adnexectomy at the age of 49 years for benign gynaecological indications.

The third SCCOHT patient (**patient #3**) was diagnosed at the age of 35 years. The three-generation pedigree showed no further patients with BC or OC. She presented with an ovarian tumour of 14x13x9cm, ascites and pleural effusion, severe hypercalcaemia (4.23 mmol/l), and acute kidney failure grade III with suppression of vitamin D and parathyroid homone. Furthermore, she was diagnosed with an adrenogenital syndrome with primary aldosteronism and adrenocortical hyperplasia. Tumour cells were confined to the inside of the ovary (corresponding to pT1a, G3, Ki67 70%), ascites showed no malignant cells. Because of the reduced general condition, a lymphonodectomy and histologic confirmation of the suspected liver metastasis were not performed (suspected FIGO IVB). She died one year after initial diagnosis, surgery and adjuvant chemotherapy with cisplatin, doxorubicin, and etoposide cyclophosphamide (PAVEP regimen). Genetic analysis of blood-derived DNA revealed no PV in any of the established OC predisposition genes. Genetic analysis of the tumour-derived DNA revealed a somatic PV (c.3168+1G>A, p.(Gly1028_Glu1056del)) in exon 22 of *SMARCA4* with a VF of 97% (read coverage: 1847), suggesting a biallelic *SMARCA4* inactivation.

**References**

FERLICOT, S., BESSOUD, B., MARTIN, V., BRANCHEREAU, S., FABIANI, B., FOURRÉ, C., BRAILLY, S., GUERIS, J., VALTEAU, D., GAUTHIER, F. & FABRE, M. 1998. [Large cell variant of small cell carcinoma of the ovary with hypercalcemia]. *Ann Pathol,* 18**,** 197-200.

LEPKES, L., KAYALI, M., BLUMCKE, B., WEBER, J., SUSZYNSKA, M., SCHMIDT, S., BORDE, J., KLONOWSKA, K., WAPPENSCHMIDT, B., HAUKE, J., KOZLOWSKI, P., SCHMUTZLER, R. K., HAHNEN, E. & ERNST, C. 2021. Performance of In Silico Prediction Tools for the Detection of Germline Copy Number Variations in Cancer Predisposition Genes in 4208 Female Index Patients with Familial Breast and Ovarian Cancer. *Cancers (Basel),* 13.

LU, B. & SHI, H. 2019. An In-Depth Look at Small Cell Carcinoma of the Ovary, Hypercalcemic Type (SCCOHT): Clinical Implications from Recent Molecular Findings. *J Cancer,* 10**,** 223-237.

PAUTIER, P., RIBRAG, V., DUVILLARD, P., REY, A., ELGHISSASSI, I., SILLET-BACH, I., KERBRAT, P., MAYER, F., LESOIN, A., BRUN, B., CROUET, H., BARATS, J. C., MORICE, P. & LHOMME, C. 2007. Results of a prospective dose-intensive regimen in 27 patients with small cell carcinoma of the ovary of the hypercalcemic type. *Ann Oncol,* 18**,** 1985-9.

PLON, S. E., ECCLES, D. M., EASTON, D., FOULKES, W. D., GENUARDI, M., GREENBLATT, M. S., HOGERVORST, F. B., HOOGERBRUGGE, N., SPURDLE, A. B., TAVTIGIAN, S. V. & GROUP, I. U. G. V. W. 2008. Sequence variant classification and reporting: recommendations for improving the interpretation of cancer susceptibility genetic test results. *Hum Mutat,* 29**,** 1282-91.

PRAT, J. & ONCOLOGY, F. C. O. G. 2015. Staging Classification for Cancer of the Ovary, Fallopian Tube, and Peritoneum: Abridged Republication of Guidelines From the International Federation of Gynecology and Obstetrics (FIGO). *Obstet Gynecol,* 126**,** 171-4.

RICHARDS, S., AZIZ, N., BALE, S., BICK, D., DAS, S., GASTIER-FOSTER, J., GRODY, W. W., HEGDE, M., LYON, E., SPECTOR, E., VOELKERDING, K. & REHM, H. L. 2015. Standards and guidelines for the interpretation of sequence variants: a joint consensus recommendation of the American College of Medical Genetics and Genomics and the Association for Molecular Pathology. *Genet Med,* 17**,** 405-24.

WAPPENSCHMIDT, B., HAUKE, J., FAUST, U., NIEDERACHER, D., WIESMÜLLER, L., SCHMIDT, G., GROß, E., GEHRIG, A., SUTTER, C., RAMSER, J., RUMP, A., ARNOLD, N. & MEINDL, A. 2020. Criteria of the German Consortium for Hereditary Breast and Ovarian Cancer for the Classification of Germline Sequence Variants in Risk Genes for Hereditary Breast and Ovarian Cancer. *Geburtshilfe Frauenheilkd,* 80**,** 410-429.

YOUNG, R. H., OLIVA, E. & SCULLY, R. E. 1994. Small cell carcinoma of the ovary, hypercalcemic type. A clinicopathological analysis of 150 cases. *Am J Surg Pathol,* 18**,** 1102-16.
